# HIV Testing and Service Delivery Among Blacks or African Americans — 61 Health Department Jurisdictions, United States, 2013

**Published:** 2015-02-06

**Authors:** Puja Seth, Tanja Walker, NaTasha Hollis, Argelia Figueroa, Lisa Belcher

**Affiliations:** 1 Division of HIV/AIDS Prevention, National Center for HIV/AIDS, Viral Hepatitis, STD, and TB Prevention, CDC

In the United States, approximately 1.2 million persons are living with human immunodeficiency virus (HIV), of whom approximately 14.0% have not received a diagnosis. Some groups are disproportionately affected by HIV, such as persons who self-identify as blacks or African Americans (in this report referred to as blacks). Blacks accounted for 12.0% of the United States’ population but accounted for 41.0% of persons living with HIV in 2011 (1). HIV testing is critical to identify those who are infected and link them to HIV medical care for their own health and to reduce transmission to partners (2,3). To assess progress toward increasing HIV testing and service delivery among blacks in 2013, CDC analyzed national-level program data submitted by 61 health departments* and 151 directly funded community-based organizations through the National HIV Prevention Program Monitoring and Evaluation system. This report describes the results of that analysis, which found that, in 2013, blacks accounted for 45.0% of CDC-funded HIV testing events (TEs)† and more than half (54.9%) of all newly identified HIV-positive persons (in this report referred to as new positives). Among blacks, gay, bisexual, and other men who have sex with men (collectively referred to as MSM) had the highest percentage of new positives (9.6%). Broader implementation of routine HIV screening and HIV testing targeted towards populations at high risk can help identify persons with undiagnosed HIV infection and link these persons to HIV medical care and prevention services. Linkage to medical care and referrals to HIV partner services and HIV prevention services among blacks could be improved.

In 2013, CDC funded 61 health departments and 151 community-based organizations to provide HIV testing and HIV-related services in the United States. National HIV Prevention Program Monitoring and Evaluation data for CDC-funded HIV TEs and other HIV program activities are collected locally using a CDC-provided semi-standard template. Data are submitted without personal identifiers through a secure, online, CDC-supported system. CDC uses these data for monitoring and evaluation of HIV testing and HIV-related service delivery.

Valid HIV TEs were defined as tests for which either a test technology (e.g., conventional, rapid, nucleic acid amplification, or other testing) or test result (positive, negative, indeterminate, or invalid) was reported. Persons who tested HIV-positive but did not report a previous HIV-positive test result were categorized as new positives. HIV service delivery among these persons included linkage to HIV medical care (i.e., attendance at first medical appointment), referral and interview for partner services (i.e., to help persons living with HIV notify sex and drug-injecting partners of possible HIV exposure, to offer services that can protect the health of partners, and to prevent sexually transmitted disease reinfection) (4), and referral to HIV prevention services (i.e., services or interventions directly aimed at reducing the risk for transmitting or acquiring HIV) (5).

Analyses included data submitted to CDC as of June 2, 2014, which were stratified by age, sex, U.S. Census region, and selected target populations (MSM, heterosexual males, and heterosexual females).§ The percentage of missing data ranged from 8.8% to 32.8% across several service delivery indicators. CDC requires target population data for all HIV TEs in non–health care settings and only for HIV-positive TEs in health care settings (N = 424,497).¶**

In 2013, CDC funded 3,343,633 HIV TEs in the United States. Blacks accounted for 45.0% (1,506,016) of all CDC-funded HIV TEs, the largest proportion of any racial/ethnic group. Blacks accounted for 51.5% and 47.1% of TEs among all persons aged 13–19 and 20–29 years, respectively. They also accounted for 47.0% of TEs among females and 52.5% of TEs in the South. Finally, among target populations, 23.3% of TEs among MSM were among black MSM, and 52.4% and 52.0% of TEs among heterosexual males and females, respectively, were among blacks (Table 1).

Of the 1,506,016 TEs among blacks, most were among persons aged 20–29 years (42.5%) and persons living in the South (66.1%). More females (52.7%) were tested than males (46.9%). Of the 424,497 TEs with target population information, heterosexual females accounted for 32.1%. MSM and heterosexual males accounted for 8.8% and 28.1%, respectively (Table 2).

Among CDC-funded TEs in 2013, blacks accounted for 54.9% of all new positives. Blacks accounted for 68.9% and 57.9% of new positives among all persons aged 13–19 and 20–29 years, respectively. They also accounted for 68.9% of new positives among women and 65.8% of new positives in the South. Among target populations, 45.2% of new positive MSM were black, and 71.6% and 70.2% of new positive heterosexual males and females, respectively, were black (Table 1).

New positives accounted for 0.64% (9,571 of 1,506,016) of TEs among blacks. Among blacks, the HIV positivity for new positives was highest among MSM (9.6%). Although MSM accounted for 8.8% (37,222 of 424,497) of HIV TEs among blacks, they accounted for 37.3% (3,570 of 9,571) of new positive blacks. Among new positive blacks, 53.5% were linked to medical care within any timeframe after their HIV diagnosis; 44.5% were linked to medical care within 90 days; 65.8% were referred to HIV partner services; 46.4% were interviewed for HIV partner services, and 53.6% were referred to HIV prevention services. HIV service delivery was generally comparable by age group and sex, but the Midwest and South lagged in HIV service delivery. Overall, a higher percentage of new positive black MSM than heterosexual males and females were linked to HIV medical care, referred to and interviewed for HIV partner services, and referred to HIV prevention services (Table 2).

## Discussion

Blacks are disproportionately affected by HIV. In 2011, blacks accounted for 41% of all persons living with HIV in the United States (1). In 2012, the rate of HIV diagnoses was 58.3 per 100,000 for blacks, in comparison with 18.5 for Hispanics and 6.7 for whites (6). However, a national survey indicated that the percentage of blacks who had ever been tested increased from 57.0% during 2003–2006 to 64.0% during 2007–2010 and was highest among blacks during both periods when compared with other racial/ethnic groups (7). The current findings indicate that among CDC-funded HIV TEs, blacks accounted for 45.0% of HIV TEs and over half (54.9%) of all new positives in 2013. Although 8.8% of the HIV TEs among blacks were conducted among MSM, they accounted for 37.3% of all new positive blacks.

HIV testing and knowledge of HIV status are the gateway to important prevention services, and for HIV-positive persons, services along the HIV continuum of care. Early initiation and adherence to antiretroviral therapy has substantial medical benefits for HIV-positive persons and prevention benefits by reducing HIV transmission to HIV-negative partners up to 96% (2,3). Therefore, in addition to identifying new HIV-positive persons, it is critical to ensure all HIV-positive persons are linked to HIV medical care and receive necessary HIV prevention services. The National HIV/AIDS Strategy (8) has a goal for 2015 that 85.0% of persons newly diagnosed with HIV are linked to HIV medical care within 90 days of diagnosis. The current finding of 44.5% for linkage within 90 days suggests that linkage among blacks needs to be significantly improved to meet the National HIV/AIDS Strategy goal. Because rates of referrals to HIV partner services and HIV prevention services ranged from 46.4% to 65.8%, referrals to these services also could be improved.

The findings in this report are subject to at least five limitations. First, because of missing data, the service delivery data are an underestimate and represent the minimum percentage achieved, particularly for linkage to care. Second, data for target populations are only required in non–health care settings and for TEs resulting in an HIV-positive result in health care settings (28% of TEs among blacks). Therefore, results are underreporting the number of TEs that are being conducted among these populations. Third, the percentage of missing data (19.6%) is high among target populations. Fourth, because this report focuses only on some CDC-funded HIV TEs and does not represent all HIV tests conducted in the United States, these findings might not be generalizable to the entire United States. Finally, because self-report was used to identify a new HIV diagnosis, the number of new positives reported likely represents an overestimation of new positives. Given the importance of programmatic data for effective public health monitoring and evaluation, continued technical assistance is needed to help grantees improve the completeness and accuracy of data.

What is already known on this topic?

Blacks aged 18–64 years were tested more frequently for human immunodeficiency virus (HIV) than Hispanics or whites in the past 12 months. However, about 31.0% have never been tested, and 15.0% of blacks living with HIV do not know they are infected. Undiagnosed HIV infection can significantly influence HIV transmission rates in communities. In 2011, an estimated 73,600 HIV-positive blacks living in the United States were unaware of their HIV status.

What is added by this report?

An analysis of national-level program data on HIV testing and service delivery for blacks in 2013 submitted through the National HIV Prevention Program Monitoring and Evaluation system showed that blacks accounted for 45.0% of CDC-funded HIV testing events and over half (54.9%) of all newly identified HIV-positive persons. Also, 9.6% of black men who have sex with men receiving a CDC-funded test were newly identified as HIV-positive in 2013.

What are the implications for public health practice?

Linkage to medical care and referrals to HIV partner services and other HIV prevention services among blacks who obtain HIV testing services could be improved.

Continued efforts to expand routine screening as recommended by the U.S. Preventive Services Task Force (9) and CDC guidelines (10) and to target HIV testing services toward populations at high risk, such as MSM, can help identify HIV-positive persons whose infection is undiagnosed, particularly in jurisdictions with the highest HIV prevalence among blacks. Programmatic efforts to increase prevention efforts among HIV-negative persons also are critical to reduce their risk for HIV infection. Finally, linkage to care and behavioral prevention activities for HIV-positive persons are critical to ensure receipt of key services to improve their health and to prevent HIV transmission to their partners (5).

## Figures and Tables

**TABLE 1:** Number and percentage of HIV testing events and newly identified HIV-positive persons among blacks or African Americans, in comparison with all CDC-funded HIV testing events, by selected characteristics — United States, Puerto Rico, and the U.S. Virgin Islands, 2013*

Characteristic	HIV testing events			Newly identified HIV-positive persons		
	
	All CDC-funded HIV testing events	HIV testing events among blacks		All newly identified HIV-positive persons	Newly identified HIV-positive blacks	
	
		No.	(%)		No.	(%)
**Age group (yrs)**						
13–19	279,412	143,797	(51.5)	579	399	(68.9)
20–29	1,358,687	639,706	(47.1)	6,895	3,989	(57.9)
30–39	756,782	308,182	(40.7)	4,118	1,935	(47.0)
40–49	461,696	198,277	(42.9)	3,056	1,530	(50.1)
≥50	456,169	207,908	(45.6)	2,434	1,488	(61.1)
**Sex**						
Male	1,632,645	706,148	(43.3)	13,976	7,224	(51.7)
Female	1,687,367	793,894	(47.0)	3,188	2,196	(68.9)
**Region**						
Northeast	596,617	245,322	(41.1)	2,562	1,294	(50.5)
Midwest	375,204	192,506	(51.3)	1,659	956	(57.6)
South	1,896,334	995,531	(52.5)	10,314	6,787	(65.8)
West	435,008	68,679	(15.8)	2,558	530	(20.7)
U.S. dependent areas	40,470	3,978	(9.8)	333	4	(1.2)
**Target population** †						
Men who have sex with men	159,560	37,222	(23.3)	7,896	3,570	(45.2)
Heterosexual males	227,758	119,403	(52.4)	2,505	1,793	(71.6)
Heterosexual females	262,154	136,205	(52.0)	2,147	1,508	(70.2)
**Total**	**3,343,633**	**1,506,016**	**(45.0)**	**17,426**	**9,571**	**(54.9)**

**Abbreviation:** HIV = human immunodeficiency virus.

**Source:** National HIV Prevention Program Monitoring and Evaluation system.

* HIV testing events were defined as tests for which either a test technology (conventional, rapid, nucleic acid amplification testing, or other) or test result (positive, negative, indeterminate, or invalid) was reported. Persons who tested HIV-positive but did not report a previous positive test result were categorized as newly identified HIV-positive persons.

† Data to identify target populations are required for all testing events conducted in non–health care settings but are only required for HIV-positive persons from health care settings. Therefore, for target populations, HIV testing events and newly identified HIV-positive persons represent data from non–health care settings but only positive testing events from health care settings (N = 995,834 for all CDC-funded testing events and N = 424,497 for blacks). Other target populations and missing data among blacks accounted for 31.0% of HIV testing events (transgender = 0.4%, persons who inject drugs = 1.1%, persons not reporting sex with male or female or injection drug use [i.e., no risk behavior] = 9.9%, and missing = 19.6%).

**TABLE 2:** Number and percentage of newly identified HIV-positive persons and HIV service delivery among newly identified HIV-positive blacks or African Americans, by selected characteristics — United States, Puerto Rico, and the U.S. Virgin Islands, 2013*

	HIV testing events among blacks†		Newly identified HIV-positive blacks		Linked to HIV medical care within any timeframe		Linked to HIV medical care within 90 days		Referred to HIV partner services		Interviewed for HIV partner services		Referred to HIV prevention services	
							
Characteristic	No.	(%)	No.	(%)	No.	(%)	No.	(%)	No.	(%)	No.	(%)	No.	(%)
**Age group (yrs)**														
13–19	143,797	(9.5)	399	(0.28)	216	(54.1)	179	(44.9)	287	(71.9)	191	(47.9)	218	(54.6)
20–29	639,706	(42.5)	3,989	(0.62)	2,142	(53.7)	1,783	(44.7)	2,744	(68.8)	1,852	(46.4)	2,207	(55.3)
30–39	308,182	(20.5)	1,935	(0.63)	1,055	(54.5)	873	(45.1)	1,252	(64.7)	903	(46.7)	1,011	(52.2)
40–49	198,277	(13.2)	1,530	(0.77)	777	(50.8)	647	(42.3)	916	(59.9)	668	(43.7)	725	(47.4)
≥50	207,908	(13.8)	1,488	(0.72)	795	(53.4)	653	(43.9)	899	(60.4)	663	(44.6)	765	(51.4)
**Sex**														
Male	706,148	(46.9)	7,224	(1.02)	3,858	(53.4)	3,200	(44.3)	4,787	(66.3)	3,333	(46.1)	3,927	(54.4)
Female	793,894	(52.7)	2,196	(0.28)	1,194	(54.4)	1,001	(45.6)	1,405	(64.0)	1,049	(47.8)	1,132	(51.5)
**Region**														
Northeast	245,322	(16.3)	1,294	(0.53)	844	(65.2)	746	(57.7)	947	(73.2)	681	(52.6)	977	(75.5)
Midwest	192,506	(12.8)	956	(0.50)	369	(38.6)	329	(34.4)	610	(63.8)	356	(37.2)	505	(52.8)
South	995,531	(66.1)	6,787	(0.68)	3,562	(52.5)	2,867	(42.2)	4,308	(63.5)	3,057	(45.0)	3,337	(49.2)
West	68,679	(4.6)	530	(0.77)	343	(64.7)	316	(59.6)	430	(81.1)	344	(64.9)	305	(57.5)
U.S. dependent areas	3,978	(2.6)	4	(0.10)	3	(75.0)	3	(75.0)	3	(75.0)	3	(75.0)	2	(50.0)
**Target population** §														
Men who have sex with men	37,222	(8.8)	3,570	(9.6)	2171	(60.8)	1,970	(55.2)	2,821	(79.0)	2,008	(56.2)	2,417	(67.7)
Heterosexual males	119,403	(28.1)	1,793	(1.5)	914	(51.0)	814	(45.4)	1,270	(70.8)	915	(51.0)	966	(53.9)
Heterosexual females	136,205	(32.1)	1,508	(1.1)	870	(57.7)	802	(53.2)	1,126	(74.7)	844	(56.0)	892	(59.2)
**Total**	**1,506,016**		**9,571**	**(0.64)**	**5,121**	**(53.5)**	**4,261**	**(44.5)**	**6,298**	**(65.8)**	**4,441**	**(46.4)**	**5,126**	**(53.6)**

**Abbreviation:** HIV = human immunodeficiency virus.

**Source:** National HIV Prevention Program Monitoring and Evaluation system.

* The denominator for the percentage for newly identified HIV-positive persons is HIV testing events. The denominator for the percentages of all other columns is newly identified HIV-positive persons.

† The percentages for HIV testing events are column percentages. For target populations, the denominator is 424,497, and for all other client characteristics, the denominator is 1,506,016.

§ Not required for persons who test negative in health care settings. Data to identify target populations are required for all testing events conducted in non–health care settings but are only required for HIV-positive persons from health care settings. Therefore, for target populations, HIV testing events and newly identified HIV-positive persons represent data from non–health care settings but only positive testing events from health care settings (N = 424,497). Other target populations and missing data accounted for 31.0% of HIV testing events (transgender = 0.4%, persons who inject drugs = 1.1%, persons not reporting sex with male or female or injection drug use [i.e., no risk behavior] = 9.9%, and missing = 19.6%).

## Footnotes

* Grantees include health departments in the 50 states, the District of Columbia, Puerto Rico, the U.S. Virgin Islands, and eight directly funded city/county health departments (Baltimore, Maryland; Chicago, Illinois; Fulton County, Georgia; Houston, Texas; Los Angeles County, California; New York, New York; Philadelphia, Pennsylvania; and San Francisco, California).

† An HIV testing event is one or more HIV tests performed to determine a person’s HIV status. During one TE, a person might be tested once (e.g., one rapid test or one conventional test) or multiple times (e.g., one rapid test followed by one conventional test to confirm a preliminary HIV-positive test result). Valid HIV TEs were defined as tests for which either a test technology (conventional, rapid, nucleic acid amplification, or other testing) or test result (positive, negative, indeterminate, or invalid) was reported.

§ MSM are males who reported male-to-male sexual contact in the past 12 months. Heterosexual males are males who only reported contact with a female in the past 12 months. Heterosexual females are females who only reported contact with a male in the past 12 months.

¶ A health care setting is defined as a site that provides medical diagnostic and treatment services (e.g., inpatient facilities, outpatient facilities, and emergency departments). A non–health care setting is a site that does not provide medical diagnostic and treatment services (e.g., HIV counseling and testing sites and community settings).

** Other target populations and missing data among blacks accounted for 31.0% of HIV testing events (transgender = 0.4%, persons who inject drugs = 1.1%, persons not reporting sex with male or female or injection drug use (i.e., no risk behavior) = 9.9%, and missing = 19.6%).
